# Integrative genomic and multi-omics analyses identify oxidative stress-related pathways and druggable targets for tinnitus

**DOI:** 10.3389/fneur.2026.1775859

**Published:** 2026-04-28

**Authors:** Jingjie Han, Ying Cao, Cai Zhang, Qianqian Zhang, Jinying Li, Hongen Xu, Xingle Zhao, Changyun Yu

**Affiliations:** 1 Country Department of Otolaryngology Head and Neck Surgery, The First Affiliated Hospital of Zhengzhou University, Zhengzhou, China; 2 Country Precision Medicine Center, Academy of Medical Sciences, Zhengzhou University, Zhengzhou, China

**Keywords:** *ACADVL*, drug target prediction, mediation analyses, multi-omics integration, oxidative stress, tinnitus

## Abstract

**Background:**

Tinnitus is a complex auditory perceptual disorder often accompanied by neuroinflammatory responses and metabolic abnormalities. Increasing evidence suggests that persistent oxidative stress, together with its interactions with immune regulation and energy metabolism, contributes to the pathophysiology of tinnitus. However, the molecular mechanisms by which oxidative stress drives tinnitus development remain to be systematically elucidated.

**Methods:**

We integrated GWAS meta-analysis with DNA methylation, gene expression, and protein QTLs for 1,844 oxidative stress-related genes. Candidate genes were prioritized via SMR and colocalization, then validated in independent cohorts and brain-specific datasets, and further experimentally confirmed by assessing protein and RNA levels in human peripheral blood plasma. To further investigate oxidative stress-related pathways, mediation analyses were conducted, while molecular docking explored druggability.

**Results:**

Based on integrated multi-omics evidence, *ACADVL* was identified as a primary candidate target for tinnitus. Beyond *in silico* analyses, compared with healthy controls, *ACADVL* mRNA expression and *VLCAD* protein levels were both increased in patients with tinnitus. *ACADVL* increased tinnitus risk by suppressing CD25 on IgD^+^CD38^−^ B cells (10%) and altering the phosphate-to-oleoyl-linoleoyl-glycerol ratio (8.5%), highlighting oxidative stress -mediated immune and metabolic pathways. Molecular docking confirmed fenretinide as a potential therapeutic agent.

**Conclusion:**

This study provides convergent evidence from genetic, multi-omics, and experimental analyses that oxidative stress–related genes, particularly *ACADVL*, may increase susceptibility to tinnitus through metabolic and inflammatory dysregulation. Molecular docking and drug enrichment analyses further confirmed the druggability of these targets, highlighting fenretinide as a promising repurposable therapeutic candidate. By integrating genetic epidemiology, functional validation, and drug target identification based on molecular docking, this work establishes a framework for mechanistic investigation and therapeutic development in tinnitus.

## 1 Introduction

Tinnitus is increasingly recognized as a symptom of underlying conditions rather than an independent disorder (1, 2). It is defined as the perception of sound in the absence of an external acoustic stimulus (3, 4). Globally, tinnitus affects more than 740 million individuals, with over 120 million reporting severe health impacts, and its prevalence rises markedly with age (5). Beyond the auditory symptom itself, tinnitus is frequently associated with psychiatric and neurological comorbidities (6) such as anxiety, depression (7), insomnia (8, 9), and migraine (10). These comorbidities significantly impair quality of life and may even increase suicide risk (11). Despite its widespread prevalence and heavy burden, the molecular mechanisms of tinnitus remain unclear, and current interventions are primarily symptomatic, lacking precision strategies that target specific molecular pathways (12).

Oxidative stress (OS) is characterized by an imbalance between the production of reactive oxygen species (ROS) and endogenous antioxidant defense (13). The cochlea, being highly metabolically active, is particularly susceptible to ROS-related injury (14), leading to damage of hair cells and synapses and, subsequently, triggering neuroinflammation and excitotoxicity (15). Evidence from experimental models has demonstrated enrichment of OS-related pathways in tinnitus (16, 17), while clinical studies suggest systemic redox imbalance in patients (18, 19). Furthermore, antioxidant interventions (20) have been shown to reduce oxidative damage, alleviate ototoxicity, and even partially reverse pathological changes (21), highlighting the central role of OS in tinnitus pathophysiology.

In recent years, the contribution of genetic factors to tinnitus has been increasingly recognized. According to the multidisciplinary framework proposed by Dirk De Ridder and colleagues (22), a conceptual distinction has been made between tinnitus and tinnitus disorder. In contrast to tinnitus, tinnitus disorder involves additional emotional and cognitive networks beyond auditory perception. These two conditions may also differ in their genetic architecture: tinnitus appears to be primarily influenced by common genetic variants, whereas tinnitus disorder is more likely to be associated with rare variants of larger effect. Despite these advances, the functional interpretation of genetic findings in tinnitus remains limited. Due to restricted access to human brain tissue and ethical constraints, most evidence comes from animal models (23). Genetic approaches provide an alternative means to investigate the molecular basis of tinnitus at the population level. Genome-wide association studies (GWAS) have identified several tinnitus susceptibility loci (24), highlighting the role of common variants in disease risk and revealing genetic correlations with hearing loss, depression, and other neuropsychiatric conditions (25). Furthermore, Meta-analysis across multiple cohort GWAS datasets can substantially improve statistical power, reveal novel loci, and refine the genetic architecture of tinnitus (26). In parallel, quantitative trait locus (QTL) analyses, including methylation (mQTL), expression (eQTL), and protein (pQTL), provide a multi-omics framework to bridge genetic variation with molecular phenotypes (27, 28). Such integrative approaches provide complementary insights across biological layers (29, 30), improving our ability to elucidate the fundamental mechanisms underlying complex traits such as tinnitus.

Building on this background, we used meta-analyzed GWAS data integrated with cis-mQTL, cis-eQTL, and cis-pQTL datasets to identify OS-related genes associated with tinnitus and validated them in brain tissue eQTL data. Multi-omic integration enabled gene prioritization, mediation analysis of metabolic and immune pathways, and construction of a molecular network linking oxidative stress to tinnitus. Finally, drug target prediction and molecular docking provided genetic support for potential therapeutic strategies.

## 2 Materials and methods

### 2.1 Study design

**Figure 1:**
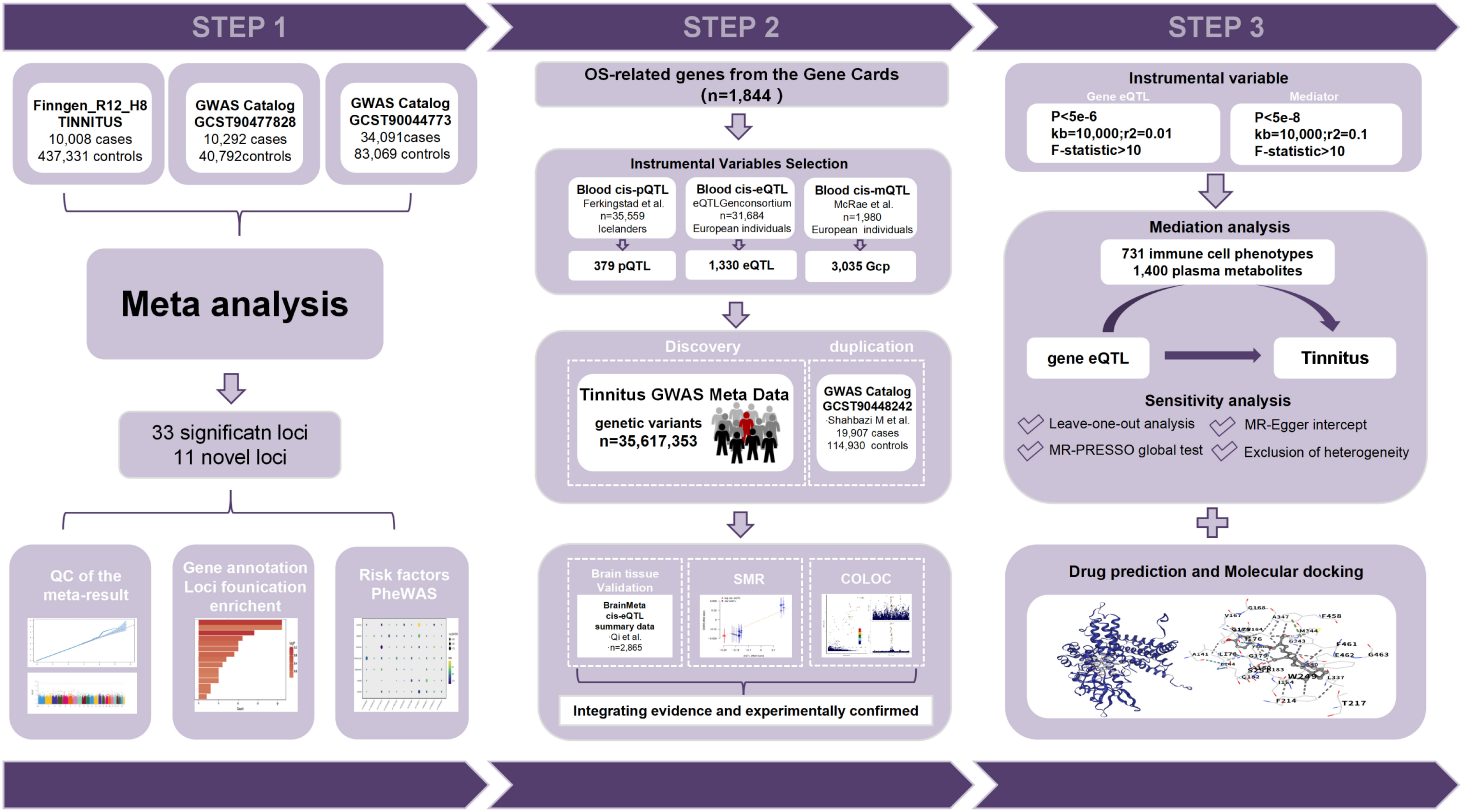
Study workflow for identifying OS-related genes associated with tinnitus and subsequent functional analyses.

The overall framework of this study is illustrated in Figure 1. Publicly available cis-QTL and GWAS summary data were integrated to explore associations between oxidative stress-related genes and tinnitus. A meta-analysis of three independent GWAS datasets was followed by gene annotation, enrichment, and risk factor reliability assessment. SMR and colocalization analyses were then performed across DNA methylation, gene expression, and protein abundance layers. Key genes were validated in independent cohorts and by cross-tissue comparison using brain-specific eQTL and mQTL data, and the primary target was further experimentally confirmed. Bidirectional Mendelian randomization and mediation analyses established causal and regulatory relationships, while molecular docking predicted potential therapeutic targets.

### 2.2 Data source

Detailed information for each variable is provided in Supplemental Table S1.

#### 2.2.1 Tinnitus datasets

In the discovery stage, GWAS summary statistics were derived from a meta-analysis of three independent cohorts. The first dataset originated from the Finnish FinnGen project (https://www.finngen.fi/en), which defines phenotypes using nationwide health registry data, including hospital diagnoses and prescription records (31). In FinnGen, tinnitus (H8_TINNITUS) was defined based on registry-derived ICD codes (ICD-10 H93.1 and ICD-9 388.3). This dataset included 10,008 tinnitus cases and 437,331 controls. Two additional datasets were obtained from the GWAS Catalog GCST90477828 and GCST90044773 (https://www.ebi.ac.uk/gwas/). In the Million Veteran Program (MVP), an extensive longitudinal study that analyzed 2,068 traits in 635,969 participants, tinnitus was defined using PheCode 389.4 derived from ICD-9 and ICD-10 diagnostic codes in electronic health records, comprising 10,292 tinnitus cases and 40,792 controls (32). The other was derived from the UK Biobank hearing loss cohort, from which tinnitus phenotypes were extracted from self-reported questionnaire responses regarding ringing or buzzing sounds in the ears or head, comprising 34,091 cases and 83,069 controls (33). To validate the loci identified in the discovery stage, we used an independent replication cohort: a dedicated UK Biobank tinnitus GWAS including 19,907 cases and 114,930 controls (34).

#### 2.2.2 Oxidative stress-related genes

OS-related genes were identified in the GeneCards database (https://www.genecards.org) using the keyword “oxidative stress”. According to previously established standards for OS-related research, we selected 1,844 genes with a relevance score ≥ 7 (35). The complete gene list is provided in Supplementary Table S2. To further support their biological relevance, the gene list was validated through GO and KEGG pathway analyses.

#### 2.2.3 . Comorbidities and risk factors

GWAS summary statistics for smoking, alcohol consumption, sensorineural hearing loss, migraine, and insomnia were obtained from the FinnGen project (https://www.finngen.fi/en). Summary statistics for anxiety and depression were obtained from the Psychiatric Genomics Consortium (PGC, https://pgc.unc.edu/), a global collaborative initiative focusing on the genetic architecture of psychiatric disorders. To evaluate the potential associations between the tinnitus-associated loci identified in our meta-analysis and these risk factors, we extracted the corresponding SNPs from the publicly available GWAS summary statistics for each trait. For each risk factor, SNPs overlapping with our tinnitus loci were selected, and their effect sizes (beta), standard errors, and p-values were retrieved. These data were then combined and visualized as a bubble plot to illustrate the strength and direction of associations across traits. Multiple testing correction (FDR) was applied to provide a conservative assessment.

#### 2.2.4 . QTL datasets

The Blood pQTL data were obtained from a study of 35,559 Icelandic individuals that analyzed 4,907 proteins and identified 18,084 pQTLs (36). Blood eQTL data were retrieved from the eQTLGen Consortium (https://eqtlgen.org/cis-eqtls.html), which combined 31,684 European blood samples to assess genetic regulation of gene expression systematically (37). Blood mQTL data were based on a meta-analysis of two European cohorts (LBC_BSGS_meta, n = 1,980)(38). For validation, brain eQTL data were derived from 2,443 European individuals with genome-wide SNP data (39), and brain mQTL data were derived from a meta-analysis of three European cohorts (Brain-mMeta, n = 1,160) (40). To minimize potential bias due to horizontal pleiotropy, all QTL analyses were restricted to the cis-acting region.

#### 2.2.5 . Metabolites and immune cell traits

Metabolite data were obtained from a large metabolomics GWAS of the CLSA, which included 8,299 individuals and reported genetic associations for 1,091 metabolites and 309 metabolite ratios (41). These data are accessible from the GWAS Catalog database, with identifiers spanning from GCST90199621 to GCST90201020 (https://www.ebi.ac.uk/gwas/). GWAS summary statistics for 731 immune cell traits were also downloaded from the GWAS Catalog, GCST90001391 to GCST90002121, covering 3,757 European individuals (42).

### 2.3 . Methods

#### 2.3.1 . Summary-data-based Mendelian randomization (SMR)

We performed SMR and HEIDI tests using the SMR software (version 1.3.1) to evaluate the associations and potential pleiotropy between OS-related gene methylation, gene expression, protein abundance, and tinnitus (43). The 1,000 Genomes Project European panel was used as the reference dataset (https://www.internationalgenome.org/). Analyses included cis-QTLs within 1,000 kb of the target gene that reached genome-wide significance (*P* < 5.0 × 10^−8^). SNPs with allele frequency discrepancies greater than 0.2 between the reference and summary data were excluded (44). HEIDI heterogeneity testing was conducted to distinguish pleiotropy from linkage. Only SNPs passing both P_SMR < 0.05 and P_HEIDI > 0.05 were retained, indicating that the observed associations were unlikely due to linkage disequilibrium (45).

#### 2.3.2 . Colocalization analysis

We applied the Coloc R package (version 5.2.3) to determine whether two traits share the same causal variant within a given genomic region (46, 47). Analyses included SNPs within ± 1,000 kb of the lead SNP, using QTL data as the exposure and GWAS data as the outcome (48). Five posterior probabilities were calculated: H0, neither trait has a causal variant; H1, only trait 1 has a causal variant; H2, only trait 2 has a causal variant; H3, both traits have distinct causal variants; and H4, both traits share the same causal variant. Default priors were set as p1 = 10^−4^, p2 = 10^−4^, and p12 = 10^−5^. Strong colocalization evidence was defined as PPH4 / (PPH3 + PPH4) ≥ 0.7(49).

#### 2.3.3 Multi-omics evidence integration

To systematically dissect the multidimensional regulatory mechanisms of OS-related genes in tinnitus, genes were stratified according to the strength and consistency of multi-omics evidence into three confidence levels (50). Given that proteins represent the ultimate functional effectors, proteomic evidence was assigned the highest priority. High-confidence candidates demonstrated robust associations with tinnitus at both protein abundance and gene expression levels across discovery and replication cohorts, were supported by colocalization with PPH4 / (PPH3 + PPH4) ≥ 0.7, and were further validated in brain eQTL datasets. Moderate confidence candidates exhibited significant associations at both DNA methylation and gene expression levels in both cohorts, were supported by colocalization, and received additional validation from brain eQTL or mQTL data. Supportive candidates showed significant associations at either the protein abundance level or at both DNA methylation and gene expression levels across two cohorts, with colocalization evidence. This stratification highlights genes with varying levels of confidence as potential causal mediators of tinnitus through multi-layered regulatory mechanisms.

#### 2.3.4 Experimental validation of peripheral plasma expression levels in patients with tinnitus uses ELISA and RT-qPCR

To enhance the robustness of our inferred findings, additional experimental validation was conducted. The study was conducted in accordance with the Declaration of Helsinki and approved by the Institutional Review Board of The First Affiliated Hospital of Zhengzhou University (Approval No. 2025-KY-0728, June 4, 2025). Informed consent was obtained from all subjects involved in the study.

Plasma VLCAD protein levels were measured using a commercial ELISA kit (MLBIO, Shanghai, China) according to the manufacturer's instructions. All experiments were performed in triplicate. Total RNA was extracted from peripheral blood plasma samples using TRIzol reagent (Invitrogen). Reverse transcription (RT) was conducted using the HiScript^®^ III All-in-one RT SuperMix Perfect for quantitative polymerase chain reaction (qPCR) (Vazyme, Nanjing, China), according to the manufacturer's instructions. Quantitative real-time PCR was performed using Taq Pro Universal SYBR qPCR Master Mix (Vazyme, China) on a QuantStudio™ 5 Flex Real-Time PCR System (Applied Biosystems, USA). Relative gene expression levels were calculated using the 2^−^ΔΔCt method. The primer sequences used for PCR amplification were provided in Supplementary Table S3.

#### 2.3.5 Mediation analysis

Given the established roles of plasma metabolites and immune cells in oxidative stress, both were introduced as mediators in the analysis. eQTLs were used as exposures, with tinnitus as the outcome, to explore potential downstream mechanisms (51). All analyses were performed using the TwoSampleMR R package. Instrument selection adhered to the assumptions of relevance (52), independence, and exclusion restriction. To account for potential pleiotropy and heterogeneity, we conducted multiple sensitivity analyses (53), including MR-Egger regression with an intercept *P* > 0.05 indicating no pleiotropy; MR-PRESSO global and outlier assessments to detect pleiotropic effects; Cochran's Q test for heterogeneity, with *P* < 0.05 denoting significant heterogeneity; and leave-one-out analyses to evaluate the robustness of the results.

#### 2.3.6 Drug target prediction and molecular docking

We used the DSigDB database (https://dsigdb.tanlab.org/DSigDBv1.0/) to predict drugs with gene interactions and conducted enrichment analyses to evaluate the therapeutic potential of candidate genes. Chemical structures of candidate compounds were obtained from PubChem (http://pubchem.ncbi.nlm.nih.gov/), and protein structures from the PDB database (http://www.rcsb.org/). Molecular docking was performed using CB-DOCK2 (https://cadd.labshare.cn/cb-dock2/), which detects protein binding cavities via the CurPocket algorithm and conducts blind docking of ligands to potential binding sites. Docking results were visualized to show ligand conformations within binding pockets, and binding affinities were quantified using VinaScore, where lower scores indicate stronger interactions (54). Protein-ligand interactions were further analyzed using PLIP (Protein-Ligand Interaction Profiler). Three-dimensional structural visualization and figure preparation were conducted using PyMOL (version 3.0.3, Schrödinger, LLC, USA).

## 3 Results

### 3.1 Genome-wide meta-analysis identifies 11 novel loci for tinnitus

**Figure 2:**
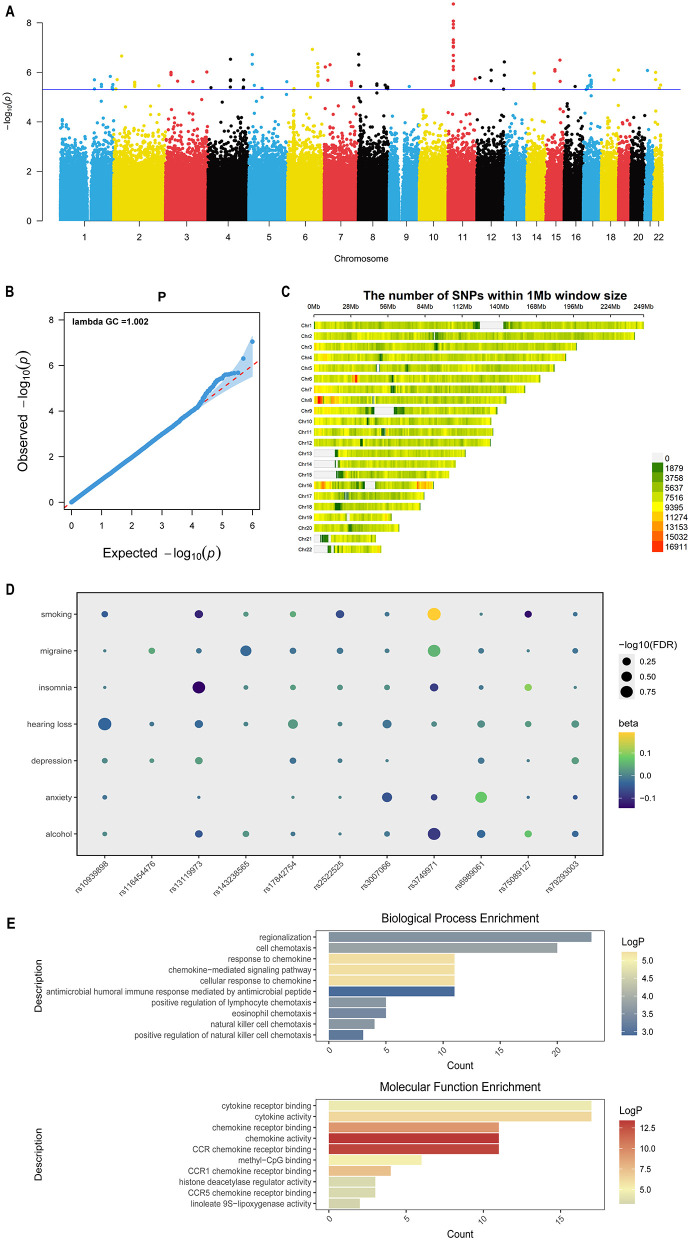
Quality control summary for the GWAS meta-analysis and gene annotation and enrichment analysis results. **(A)** Manhattan plot of the Tinnitus GWAS meta-analysis. The –log10(p) of association for all SNPs were plotted on the y-axis against genomic position on the x-axis. The blue dotted line indicates the genome-wide significance threshold (5e-06). The summary statistics of all significant SNPs were provided in Supplementary Table S3. **(B)** Quantile-Quantile (Q-Q) plot of the GWAS for tinnitus. The red line represents the identity line (y = x). The y-axis shows the observed –log_10_ (P) values, and the x-axis shows the expected –log_10_ (P) values. **(C)** SNP density plot across the genome within 1 Mb windows. The color gradient reflects the number of SNPs per window, with green indicating low density and red indicating high density. This visualization highlights regions of varying SNP coverage across chromosomes. **(D)** Bubble plot showing the associations between novel loci and seven common risk factors. Each bubble represents a single SNP, with its position reflecting the strength of association with each risk factor. Bubble size corresponds to the –log_10_ (P) value of the association, and color indicates the direction of effect. **(E)** Gene Ontology enrichment analysis, The top 10 enriched biological processes (BP) and molecular functions (MF) are shown. Bar length represents the number of genes mapped to each term (Count), and color indicates significance.

We conducted a meta-analysis by integrating two studies from the GWAS Catalog and the FinnGen tinnitus GWAS, encompassing 35,617,353 variants. A suggestive threshold of *P* < 5 × 10^−6^ was applied; the genes are listed in Supplementary Table S4. A meta-analysis Manhattan plot (Figure 2A) revealed 137 loci surpassing the genome-wide suggestive threshold, underscoring potentially biologically relevant associations with Tinnitus. Quality control and visualization demonstrated robust model performance: the QQ plot (Figure 2B) closely followed the diagonal, suggesting no systematic inflation. At the same time, the rightward deflection at the tail indicated the presence of accurate association signals. SNP density (Figure 2C) further supported the reliability of the findings. Following LD pruning of the 137 suggestive loci, 44 independent loci, including 11 novel and 33 previously reported loci, were retained for analysis, as summarized in Supplementary Table S5. The reported loci were defined as independent genomic regions identified after LD-based clumping of SNPs exceeding the suggestive threshold (*P* < 5 × 10^−6^) in each of the three independent GWAS datasets, The novel loci were defined as those located more than 500 kb away from previously reported variants. These loci were carried forward for functional annotation and downstream validation.

### 3.2 Phenotype-specific validation of novel loci

To evaluate the specificity of the novel identified loci, we examined their genetic correlations with seven common risk factors for tinnitus, including smoking, alcohol consumption, sensorineural hearing loss, migraine, insomnia, anxiety, and depression. As shown in Figure 2D and Supplementary Table S6, the bubble plot reveals that several loci show suggestive associations across multiple risk factors, indicating potential functional relevance. These observations not only support the involvement of the identified loci in diverse tinnitus-related pathways but also highlight the pronounced heterogeneity of tinnitus phenotypes.

### 3.3 Gene annotation and enrichment analysis

To investigate the biological significance of the 44 tinnitus-associated loci, we defined ± 500 kb regions around each SNP based on the GRCh37/hg19 reference genome, resulting in 571 unique genes. Enrichment analysis illustrated in Figure 2E showed involvement in immune cell migration, chemotaxis, and developmental processes affecting cochlear or auditory nerve function. Molecular functions included chemokine/cytokine signaling, OS-related enzymes, and transcriptional regulation via histone deacetylases and methyl-CpG-binding proteins, suggesting these loci may influence tinnitus through combined effects on immunity, oxidative stress, and gene expression.

### 3.4 Identification of OS-related genes and cis-QTLs

From the GeneCards database, we retrieved 1,844 genes associated with OS. Subsequent enrichment analyses revealed that these genes were significantly enriched in oxidative stress-related pathways, as shown in Supplementary Figure S1. Within blood-based QTL resources, we identified 1,330 cis-eQTLs, 3,035 cis-mQTLs, and 379 cis-pQTLs corresponding to these genes.

### 3.5 Integration of QTLs with tinnitus GWAS

#### 3.5.1 cis-pQTL integration

**Figure 3:**
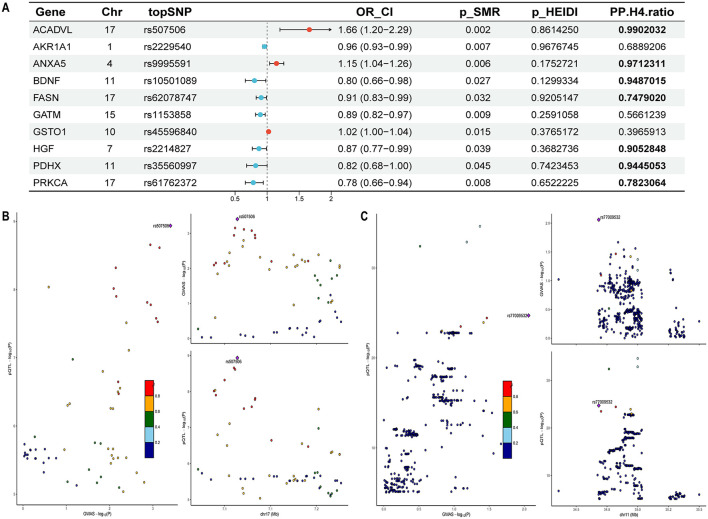
SMR and Colocalization results for the association between the protein abundance of OS-related genes and Tinnitus risk in the discovery cohort. **(A)** The forest plot of OS-related genes pQTL and Tinnitus, OR_CI, the odds ratio and confidence interval; SMR, summary data-based Mendelian randomization; HEIDI, heterogeneity in dependent instruments; PP.H4. ratio, posterior probability for hypothesis 4 (PP.H4. Ratio = PPH4 / (PP.H4 + PP.H3) > 0.7). **(B)** Regional association plots for selected loci showing SNP-level GWAS (y-axis: –log_10_ P) and ACADVL pQTL (y-axis: –log_10_ P) signals; color indicates linkage disequilibrium (r^2^) with the top SNP. **(C)** Regional association plots for selected loci showing SNP-level GWAS (y-axis: –log^10^ P) and BDNF pQTL (y-axis: –log_10_ P) signals; color indicates linkage disequilibrium (r^2^) with the top SNP.

Given that proteins serve as the ultimate effectors of gene function, we first conducted SMR analyses integrating cis-pQTLs with tinnitus GWAS data. Variants with P_SMR < 0.05 and P_HEIDI > 0.05 were deemed nominally significant. Ten nominally significant genes met these criteria (Figure 3A), For *ACADVL* (OR: 1.66, 95% CI: 1.20–2.29; PP.H4.ratio = 0.99) and *ANXA5* (OR: 1.15, 95% CI: 1.04–1.26; PP.H4.ratio = 0.97), a one standard deviation increase in expression corresponds to a 66% and 15% increase in tinnitus risk, respectively. But higher expression of *BDNF* (OR: 0.80, 95% CI: 0.66–0.98; PP.H4.ratio = 0.95), *PDHX* (OR: 0.82, 95% CI: 0.68–1.00; PP.H4.ratio = 0.94), *HGF* (OR: 0.87, 95% CI: 0.77–0.99; PP.H4.ratio = 0.91), *PRKCA* (OR: 0.78, 95% CI: 0.66–0.94; PP.H4.ratio = 0.78), and *FASN* (OR: 0.91, 95% CI: 0.83–0.99; PP.H4.ratio = 0.75) is associated with a reduced risk of tinnitus, these associations are further supported by colocalization. Among these, *ACADVL* (OR: 1.78, 95% CI: 1.09–2.89; PP.H4. ratio = 0.90) and *PDHX* (OR: 0.73, 95% CI: 0.56–0.95; PP.H4. ratio = 0.97) were consistently validated across both cohorts and exhibited strong evidence of colocalization (Figure 3B), each standard deviation increase in *ACADVL* expression was linked to a 78% higher risk of tinnitus, while the same increase in *PDHX* expression corresponded to a 27% lower risk. Comprehensive cis-pQTL SMR and colocalization results for both discovery and replication cohorts are summarized in Supplementary Tables S7–S8.

#### 3.5.2 cis-eQTL integration

**Figure 4:**
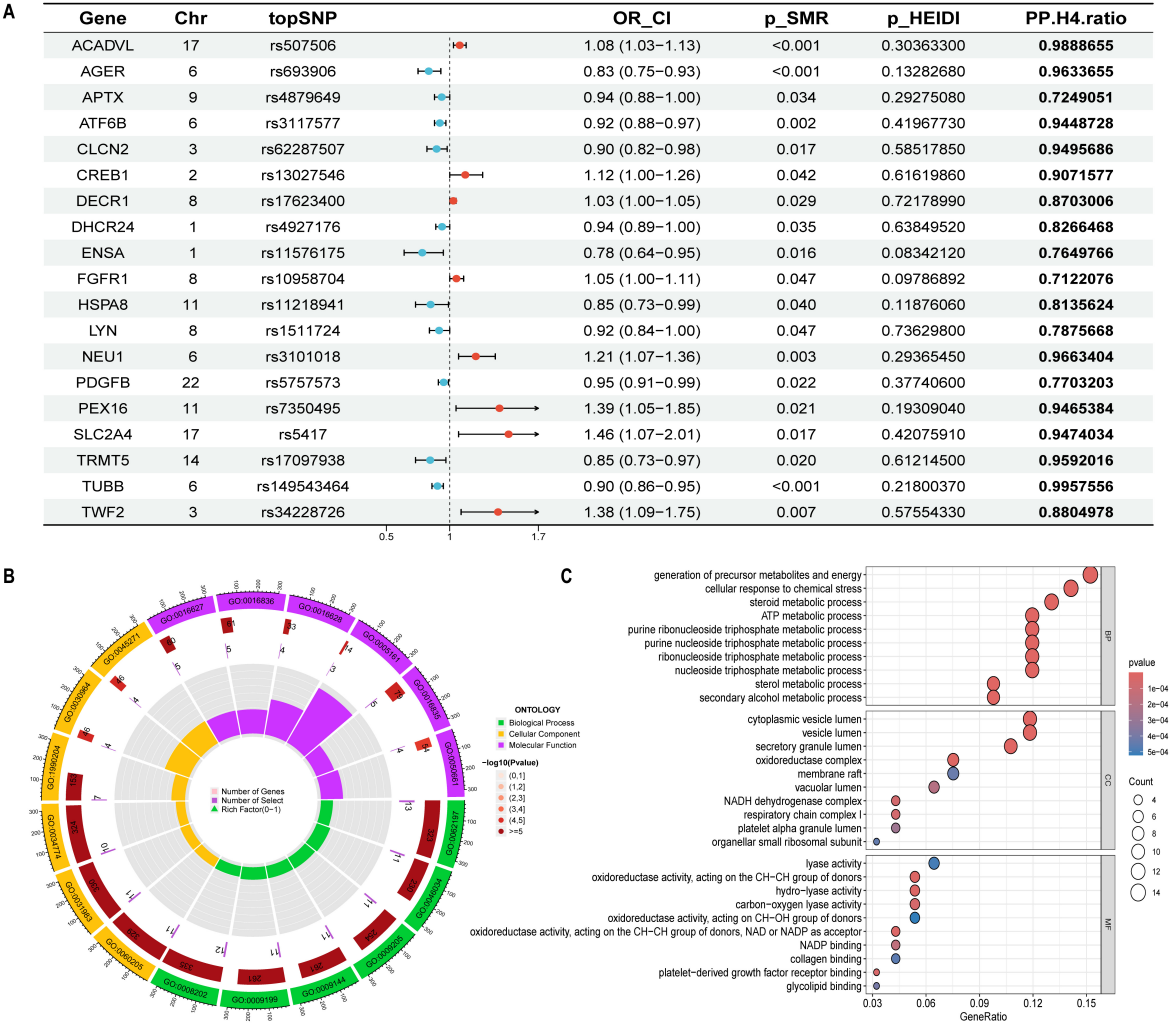
Integrative analysis of eQTLs for OS-related genes in relation to tinnitus risk. **(A)** SMR and Colocalization results for the association between the expression of OS-related genes and Tinnitus risk. OR_CI, the odds ratio and confidence interval; SMR, summary data-based Mendelian randomization; HEIDI, heterogeneity in dependent instruments; PP.H4. ratio, posterior probability for hypothesis 4, (PP.H4.Ratio = PPH4 / (PP.H4 + PP.H3) > 0.7). **(B)** Circular plot of GO enrichment analysis. Ontology categories are indicated by colored segments: green for Biological Process, yellow for Cellular Component, and purple for Molecular Function, with each segment corresponding to a GO term (ID).The second ring (red bars) represents the total number of genes annotated to each GO term, with color intensity indicating enrichment significance (–log_10_ *P*-value); darker colors denote higher significance. The third ring (purple bars) indicates the number of disease-related genes mapped to each GO term.The fourth ring represents the proportion of disease-related genes relative to the total number of genes annotated to each GO term. **(C)** Bubble plot showing GO enrichment results categorized by biological process (BP), cellular component (CC), and molecular function (MF). Dot size reflects the number of enriched gene counts, and color indicates *p*-values.

Using the same SMR and HEIDI thresholds, 93 cis-eQTLs were linked to tinnitus. Only genes supported by both colocalization and SMR in discovery and replication cohorts were considered. Detailed results are in Supplementary Tables S9–S10. The forest plot (Figure 4A) below illustrates their confidence intervals along with the colocalization ratio in the discovery cohort. GO enrichment analyses indicate that the associated genes are functionally involved in energy metabolism and redox regulation, spatially localized to mitochondrial and vesicle/membrane-related structures, and participate in cellular stress responses, metabolic homeostasis, and signal transduction through diverse enzymatic activities and molecular binding functions. These findings provide multi-level biological evidence supporting the role of OS in the pathophysiology of tinnitus (Figures 4B, C).

#### 3.5.3 cis-mQTL integration

**Figure 5:**
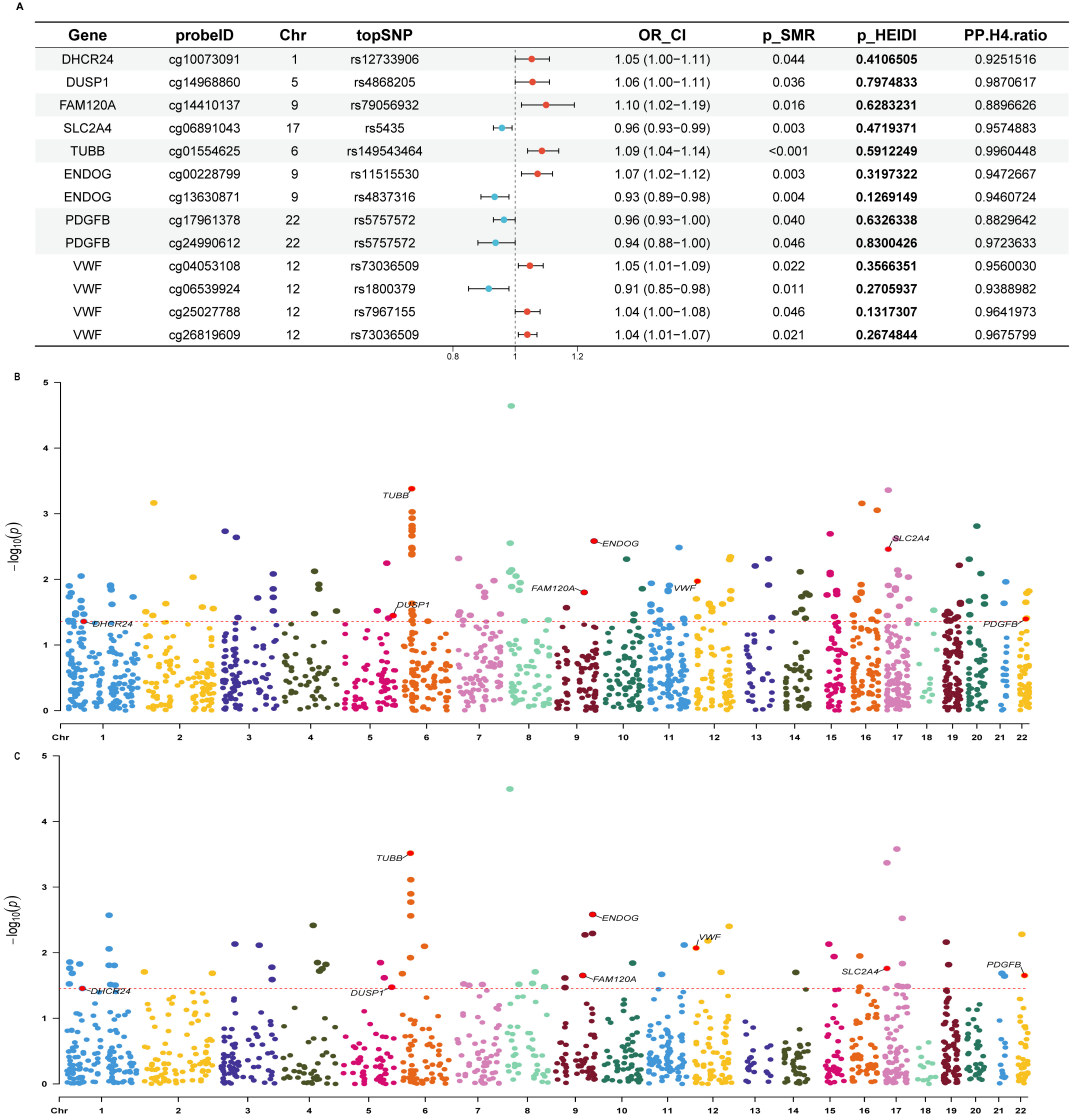
Integrative analysis of mQTLs for OS-related genes in relation to tinnitus risk. **(A)** SMR and Colocalization results for the association between the methylation sites of OS-related genes and Tinnitus risk. OR_CI, The odds ratio and confidence interval; SMR, summary data-based Mendelian randomization; HEIDI, heterogeneity in dependent instruments; PP.H4.ratio, posterior probability for hypothesis 4 (PP.H4.Ratio = PPH4 / (PP.H4 + PP.H3) > 0.7). **(B, C)** Manhattan plots of OS-related genes associated with tinnitus risk. **(B)** shows expression levels (eQTL), and **(C)** shows methylation levels (mQTL) for the same set of genes. The x-axis represents chromosome positions, and the y-axis indicates –log_10_ (p) values from SMR analysis. The dashed orange line marks the significance threshold, and red dots indicate significant genes.

We identified 75 nominally significant genes (157 cis-mQTLs) linked to tinnitus and focused on mQTLs overlapping with eQTLs. Via validation, eight candidates were highlighted (Figure 5), with replication confirming *TUBB, SLC2A4, DHCR24*, and *PDGFB* as significant at both methylation and expression levels (Supplementary Tables S11–12).

#### 3.5.4 Brain tissue validation

**Figure 6:**
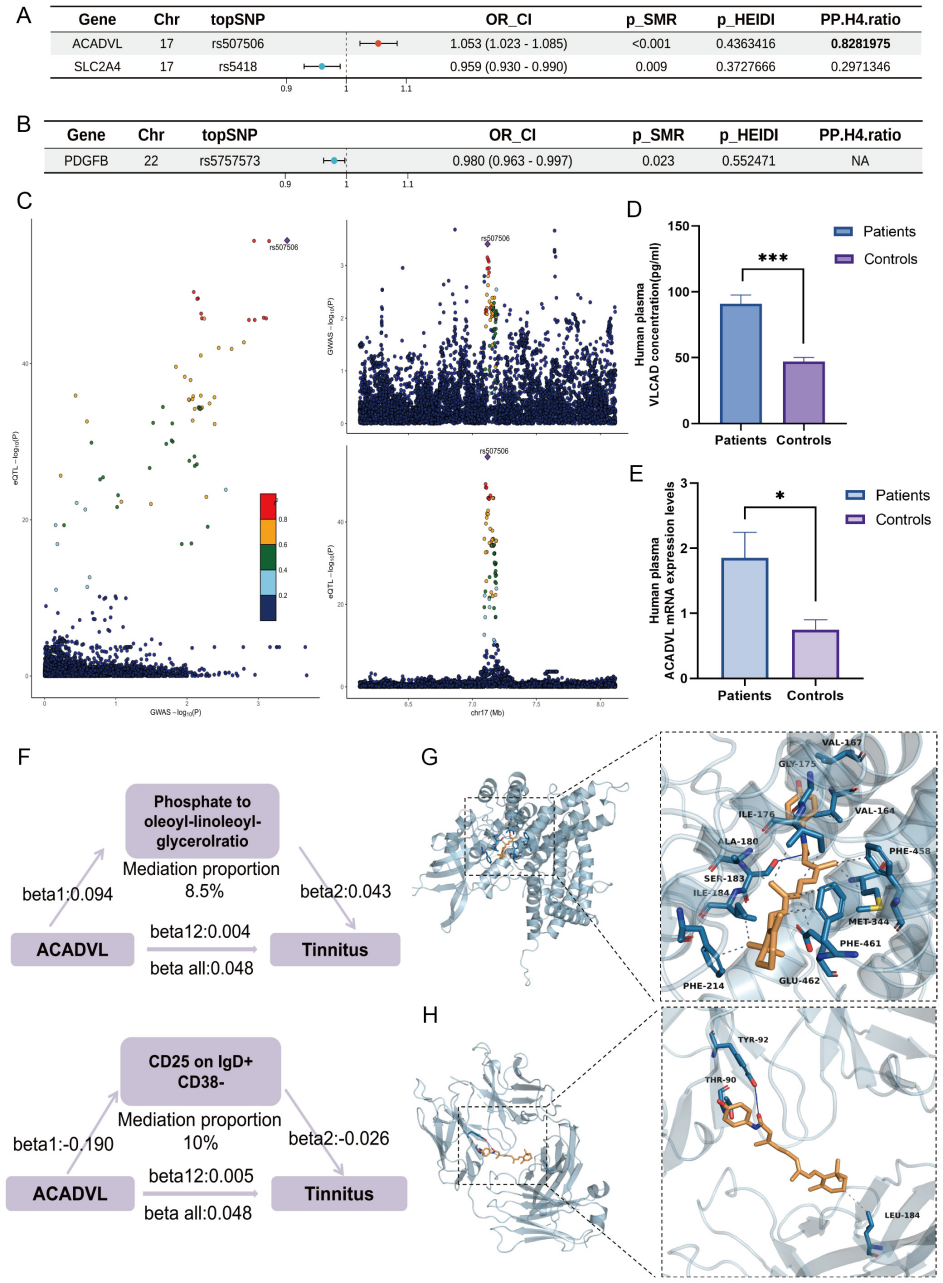
Validation using brain tissue eQTL data and experimentally confirmed,and downstream target analysis. **(A)** A forest plot about the SMR and colocalization results of ACADVL and SLC2A4 in brain tissue eQTL. **(B)** A forest plot of the SMR and colocalization results of PDGFB in brain mQTL. **(C)** The colocalization of the ACADVL genes' brain eQTL and Tinnitus GWAS positioning. Each dot represents an SNV, its color indicating LD (r)^2^, and the GWAS lead variant is displayed as a purple diamond. The genomic position (Mb) on the chromosome is shown on the X-axis, and the – log_10_ (P) of SNPs from GWAS on Tinnitus are shown on the Y-axis (top). **(D)** The ELISA results of VLCAD in Tinnitus patients and healthy controls. **(E)** The quantitative Polymerase Chain Reaction results of ACADVL in Tinnitus patients and healthy controls. **(F)** Mediation analysis model for gene expression, plasma metabolites, immune cell traits, and Tinnitus risk. beta 1: the effect of Gene expression sites on immune cell traits or plasma metabolites. beta 2: the effect of immune cell traits or plasma metabolites on Tinnitus risk. beta 12: the total effect of Gene expression on Tinnitus risk. Beta_all: the overall effect of Gene expression on the risk of Tinnitus. beta12_p: mediation proportion. **(G)** ACADVL- Fenretinide molecular docking diagrams of the main components with core molecular targets, Blue solid lines indicate hydrogen bonds, and gray dashed lines indicate hydrophobic interactions. Amino acid residues are labeled using standard amino acid abbreviations. **(H)** TUBB-Fenretinide molecular docking diagrams of the main components with core molecular targets, Blue solid lines indicate hydrogen bonds, and gray dashed lines indicate hydrophobic interactions. Amino acid residues are labeled using standard amino acid abbreviations.

Considering the pathophysiological processes underlying tinnitus, we conducted validation analyses using brain tissue eQTL and mQTL data. We also confirmed that *ACADVL* expression was associated with a 5.3% higher risk of tinnitus (OR: 1.053, 95% CI: 1.023–1.085; PP.H4 = 0.83) based on brain eQTL data, and it was further validated by colocalization analysis (Figures 6A, C). Significant expression or methylation effects of *SLC2A4* (OR: 0.959, 95% CI: 0.93–0.990) and *PDGFB* (OR: 0.980, 95% CI: 0.963–0.99) were observed in brain tissue, with each standard deviation increase in *SLC2A4* linked to a 4.1% lower risk of tinnitus and each standard deviation increase in *PDGFB* linked to a 2% lower risk (Figure 6B), but *PDGFB* lacked consistent colocalization. Summary data are in Supplementary Tables S13–14.

#### 3.5.5 Multi-omics integration

By integrating evidence across plasma eQTLs, mQTLs, pQTLs, and brain tissue QTL datasets, *ACADVL* provided the most substantial proof, being validated at plasma and brain tissue gene expression levels and plasma protein abundance in both the discovery and replication cohorts, and was therefore classified as a primary evidence gene.

### 3.6 *ACADVL* upregulation and OS–related pathways in tinnitus

As a rate-limiting enzyme involved in fatty acid β-oxidation, *ACADVL* mRNA expression and *VLCAD* protein levels in plasma were significantly increased in patients with tinnitus (Figures 6D, E). Given its established role in mitochondrial fatty acid metabolism, *ACADVL* may be linked to tinnitus through oxidative stress-related processes. The results of relative mRNA Expression of *ACADVL* and protein levels in Control and Tinnitus patients are shown in Supplementary Table S15.

### 3.7 Mediation analysis

A total of 72 metabolites and 48 immune cell traits showed significant causal associations with tinnitus. To further explore whether these traits mediate the effects of candidate genes, we performed downstream mediation analyses; all results are provided in Supplementary Figures S2–3 and Supplementary Tables S16–18.

For *ACADVL*, three immune cell traits were significantly associated, all of which increased tinnitus risk. A notable pathway revealed that *ACADVL* increased susceptibility by reducing the protective effect of CD25 on IgD^+^ CD38^−^ B cells. *ACADVL*-mediated oxidative stress may compromise CD25-related signaling within this B cell subset. Given that CD25 represents the α-chain of the interleukin-2 receptor and plays a central role in immune activation and regulation, its attenuation may disrupt immune homeostasis and promote a pro-inflammatory microenvironment. Such immune dysregulation could subsequently influence neuroimmune interactions and auditory system function, thereby contributing to tinnitus susceptibility. *ACADVL* also correlated with 11 plasma metabolites; the phosphate-to-oleoyl-linoleoyl-glycerol ratio showed a suggestive mediation effect (8.5%) (Figure 6F), while most metabolites had suppressive effects, providing mechanistic insights into how OS-related genes regulate tinnitus through oxidative stress, metabolic, and immune pathways.

### 3.8 Drug target prediction and molecular docking

Drug enrichment analyses of candidate genes identified multiple significant gene-drug associations (Supplementary Table S19). Although other compounds such as Nebivolol and cholesterol also appeared in the drug enrichment analysis, they were not prioritized for docking, considering their pharmacological profiles and docking results, as they were primarily associated with genes other than the primary target *ACADVL* (e.g., *DHCR24, PDGFB, SLC2A4*). *ACADVL* and *TUBB*, both of which were enriched for fenretinide (Fold Enrichment: 28.11, zScore: 7.28, *p* = 0.00203), providing a mechanistic rationale for its prioritization, were selected for docking. Using CB-DOCK2, five potential docking conformations were generated for each protein ligand pair. The conformation with the lowest Vina score was selected for visualization, representing the most stable interaction. Fenretinide demonstrated strong binding affinities with both targets: *ACADVL* (Vina score: −10.9 kcal/mol) and *TUBB* (Vina score: −7.9 kcal/mol). Additional results are provided in Supplementary Table S20. Fenretinide has been previously studied in oncology and metabolic disease. These pharmacological effects mechanistically align with the oxidative stress and immunometabolic pathways implicated in our study, supporting its potential repositioning as a candidate therapeutic agent. Therefore, *ACADVL* (Figure 6G) and *TUBB* (Figure 6H) emerge as core targets for future translational research and drug development in tinnitus.

## 4 Discussion

Although oxidative stress has been implicated in the pathogenesis of tinnitus, its underlying molecular mechanisms remain poorly understood. We employed an integrative multi-omics framework that systematically incorporated methylomic, transcriptomic, and proteomic layers, and combined GWAS meta-analysis with SMR and colocalization analyses to jointly identify shared genetic risk loci linking tinnitus with oxidative stress. Functional enrichment analyses demonstrated that the implicated genes were predominantly involved in inflammatory stress responses and energy metabolism pathways. Through multi-omics integration and independent validation in brain tissues, *ACADVL* was identified as a key OS-related gene in tinnitus, and its elevated expression in the peripheral blood of tinnitus patients was further experimentally confirmed. Subsequent mediation analyses were conducted to explore its potential downstream biological pathways. In addition, drug enrichment analysis and molecular docking suggested that fenretinide may have potential therapeutic value.

Within this conceptual framework, *ACADVL* was not consistently reported in previous tinnitus GWAS or rare variant studies, suggesting that it may not be detectable through single-layer genetic association approaches alone. Instead, its identification in the present study likely reflects the added value of multi-omics integration, which combines GWAS meta-analysis with cis-QTL, SMR, and colocalization analyses to prioritize genes with functional relevance across multiple regulatory layers. Therefore, *ACADVL* should be interpreted as a newly prioritized candidate gene emerging from integrative evidence rather than a replication of previously reported tinnitus susceptibility loci. Previous GWAS have primarily implicated genes involved in synaptic transmission and neuronal signaling, such as *GRM7* and *GABRA2* (26), supporting a role for excitatory–inhibitory imbalance in tinnitus. In contrast, *ACADVL* points to a distinct metabolic mechanism. As a key enzyme in mitochondrial fatty acid β-oxidation, it may influence neuronal function via energy metabolism and oxidative stress, suggesting that tinnitus may also involve upstream metabolic dysregulation. Although no direct genetic link between *ACADVL* and established tinnitus genes has been identified, a functional connection is plausible, as neuronal activity is highly energy-dependent and mitochondrial dysfunction can affect synaptic transmission and excitatory–inhibitory balance. Together, these findings extend the synapse-centered framework by introducing a metabolic dimension to tinnitus pathophysiology.

By encoding mitochondrial very long-chain acyl-CoA dehydrogenase (*VLCAD*), a rate-limiting enzyme in long-chain fatty acid β-oxidation, *ACADVL* plays a central role in mitochondrial energy metabolism. Upregulation of *ACADVL* enhances fatty acid oxidation, which, in turn, increases ROS generation and contributes to OS-related processes (55). Given the high metabolic demand and redox sensitivity of the cochlea and auditory pathways, sustained ROS accumulation may induce OS and mitochondrial dysfunction (56), processes strongly implicated in tinnitus pathophysiology. Although *ACADVL* has been primarily studied in metabolic (57, 58) and neurological disorders (59), these findings suggest that dysregulated mitochondrial energy metabolism may increase auditory tissue vulnerability. Emerging evidence highlights a close link between metabolic dysregulation and inflammatory responses. Multi-omics studies associate *ACADVL* with mitochondrial damage, inflammation, and altered glucose metabolism (58), supporting a role in immunometabolic regulation. In addition, *ACADVL* upregulation has been linked to ferroptosis and OS (60), which may mediate injury to inner ear hair cells. Under inflammatory stress, *ACADVL* can also modulate immune-related markers such as CD206 and CD62L, implicating *ACADVL* in the coordination of immune and metabolic programs. Collectively, these observations support an immunometabolic framework connecting *ACADVL* to OS, immune dysregulation, and tinnitus susceptibility. We emphasize that future experimental studies are warranted to further investigate, as suggested by our mediation analysis, how *ACADVL* affects CD25 expression and function in IgD^+^CD38^−^ B cells, as well as its potential impact on immune and metabolic activities.

Building on the identification of *ACADVL*-associated metabolic and oxidative pathways, we further explored potential pharmacological agents that could modulate these processes. Fenretinide, a synthetic retinoic acid derivative, exerts antitumor, metabolic, and immunomodulatory effects (61). It inhibits dihydroceramide desaturase, blocking *de novo* ceramide synthesis, reducing lipotoxicity, and improving mitochondrial function and cellular redox balance, thereby reducing ROS production and oxidative damage, as well as enhancing insulin signaling (62). Its antitumor mechanisms include receptor-independent ROS generation, mitochondrial permeability changes, caspase-3 activation, and apoptosis (63). Based on these mechanisms, we propose a fenretinide-tinnitus hypothesis: tinnitus is a multifactorial condition involving cochlear outer hair cell damage, mitochondrial dysfunction, oxidative stress, lipid metabolic dysregulation, and maladaptive central auditory plasticity. Accumulation of ROS and lipotoxicity exacerbates cochlear cellular stress and inflammation, disrupting auditory signal processing. Fenretinide, by modulating lipid metabolism and oxidative stress–related pathways while exerting anti-inflammatory and anti-apoptotic effects, may stabilize the cochlear and auditory neuronal microenvironment and mitigate maladaptive central hyperactivity and plasticity, thereby alleviating tinnitus symptoms. However, preclinical studies indicate that fenretinide improves hepatic steatosis and insulin sensitivity under high-fat diet conditions but may also elevate circulating ceramides and exacerbate atherosclerosis (64), highlighting the need for further preclinical and clinical evaluation of its efficacy and safety in tinnitus management. Overall, fenretinide holds promise as a candidate therapeutic agent for tinnitus, and its multi-target, multi-pathway actions provide a mechanistic rationale for translational research.

Several methodological considerations support the robustness of our findings. To account for the heterogeneity of tinnitus, we performed a GWAS meta-analysis across multiple datasets and validated the results in independent, mutually exclusive cohorts, complemented by brain tissue data. In addition, experimental validation at both RNA and protein levels in peripheral samples from tinnitus patients further supported the reliability of the key associations.

Nevertheless, several limitations should be acknowledged. Based on the current analytical framework and underlying assumptions, tinnitus has relatively low heritability and considerable heterogeneity at the individual level, which may increase the likelihood of false positive signals, particularly given the reliance on suggestive GWAS signals and nominally significant SMR results. Therefore, the identified candidate genes (including *ACADVL*) should be considered preliminary signals. Although *ACADVL* was identified as a key gene and showed upregulation in peripheral samples, particularly given the central mechanisms of tinnitus, validation in brain and cochlear tissues remains lacking and requires further validation. Incomplete annotations in lipid metabolism databases may also constrain pathway analyses. In addition, its potential as a therapeutic target also requires further biological and tissue-specific validation. Moreover, the absence of brain protein QTL data, the focus on cis-QTLs, and the restriction to European ancestry populations may limit target discovery and generalizability. GWAS meta-analysis approach used in this study is primarily designed to capture common genetic variants. However, as reported by Perez-Carpena et al. (65), the identification of common and rare variants relies on distinct methodological frameworks, with rare variant analyses typically based on gene burden tests, whereas common variant discovery is achieved through genome-wide association studies. Importantly, these approaches tend to yield partially non-overlapping sets of candidate genes. Therefore, the lack of systematic investigation of rare variants represents an additional limitation of the present study, as potentially important rare variant contributions to tinnitus susceptibility were not explored. Functional studies in cellular and animal models, such as *ACADVL* knockdown or overexpression, are required to establish direct causality before clinical translation.

In addition, the use of peripheral blood QTLs may limit direct inference of mechanisms in cochlear tissue. Highly tissue-specific oxidative stress genes, such as *NOX3*, are expressed at high levels in cochlear and vestibular sensory epithelia and spiral ganglion neurons, where they catalyze ROS production and have been implicated in acquired sensorineural hearing loss due to noise, ototoxic drugs, and aging. *NOX3* expression in the inner ear is reported to be at least 50-fold higher than in most other tissues (66), emphasizing its auditory specificity and explaining why it is unlikely to be captured in blood QTL datasets. While *NOX3* itself was not detectable in our blood-based QTL analyses, our oxidative stress gene list includes multiple *NADPH* oxidase family members and associated subunits, such as *CYBA, CYBB, NOX1, NOX4*, and *NCF1*/*NCF2*). These subunits share mechanistic pathways with *NOX3* and contribute to ROS generation in peripheral tissues (67, 68). Downstream ROS detoxification and response pathways—including *SOD1/2/3, CAT, GPX* family members, *TXN/TXNRD*, and key transcription factors such as *NFE2L2 (NRF2*) (69)—are also well represented in our dataset and functionally relevant to general oxidative stress signaling.

Taken together, these limitations suggest that while blood QTL data provide valuable insights into oxidative stress-related genes, their ability to reflect highly tissue-specific mechanisms in the inner ear remains limited. Future studies integrating multi-omics data from brain and cochlear tissues, alongside functional validation of key genes, will be essential to establish causality and support the identification of potential therapeutic targets for tinnitus.

## 5 Conclusions

This study provides convergent genetic and functional, and experimental evidence that OS-related genes, notably *ACADVL*, may contribute to tinnitus risk through metabolic and inflammatory dysregulation. These results uncover mechanistic links between redox biology and auditory dysfunction and nominate fenretinide as a promising repurposable compound. By combining genetic epidemiology, integrative multi-omics, experimental validation, and molecular docking for drug target identification, this work, although preliminary, lays a foundation for mechanistic studies and therapeutic development. Future research integrating experimental validation and clinical cohorts will be critical to evaluate safety, efficacy, and broader applicability.

## Data Availability

The original contributions presented in the study are included in the article/Supplementary material, further inquiries can be directed to the corresponding authors.
