# Supplementary material for: Integrative genomic and multi-omics analyses identify oxidative stress-related pathways and druggable targets for tinnitus
Source: Front Neurol. 2026 Apr 28;17:1775859. doi: 10.3389/fneur.2026.1775859 (PMC13160764; doi:10.3389/fneur.2026.1775859)
Supplement: Supplementary file 1 [file Supplementary_File_1.docx]

Supplementary Material

# Supplementary Figures


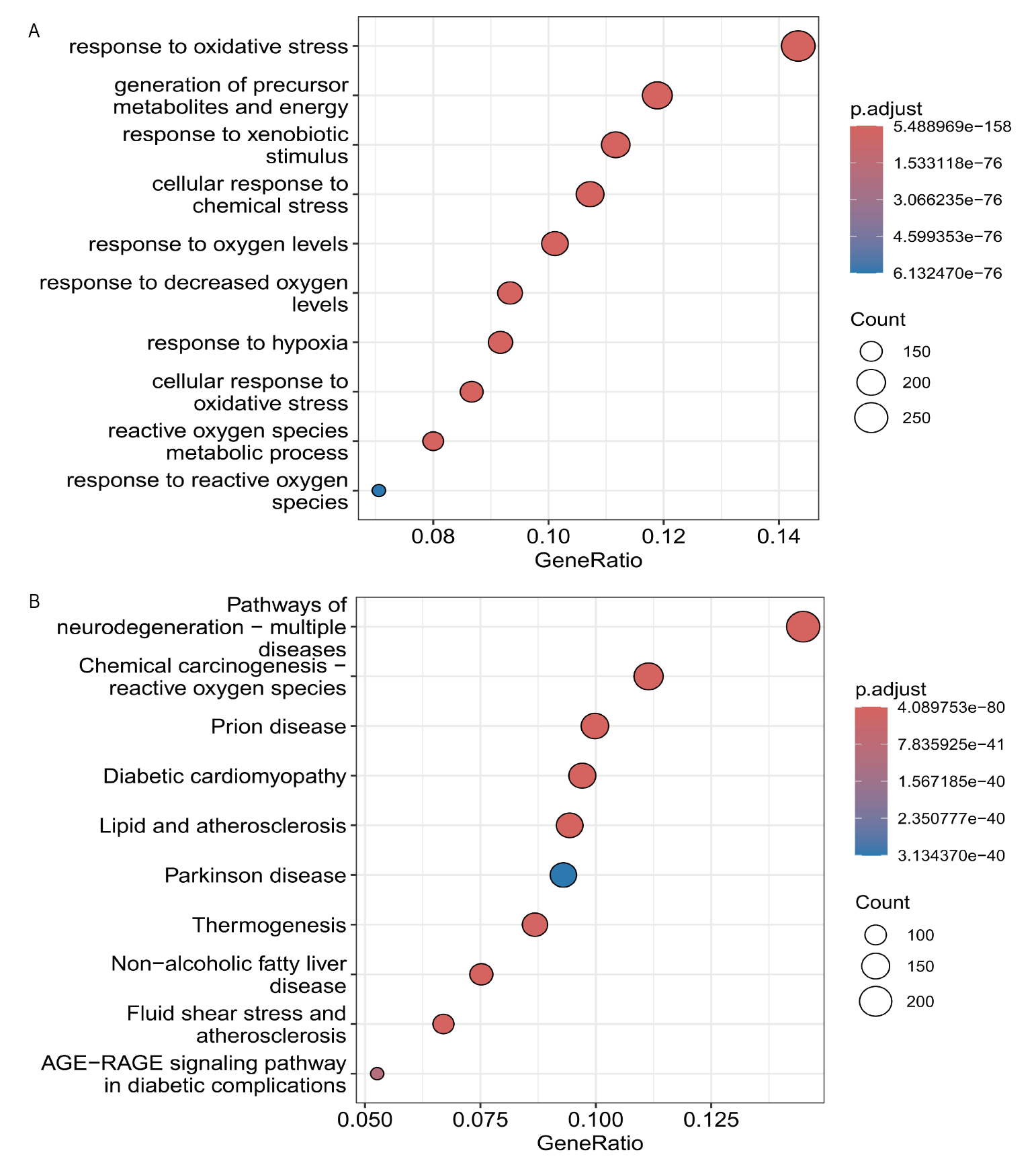


**Supplementary Figure 1. Enrichment analysis of oxidative stress–related genes (GeneCards relevance score ≥7). A.** GO analyses. **B.** KEGG analyses**.** The x-axis shows GeneRatio, bubble size indicates gene count, and color represents adjusted p-values.


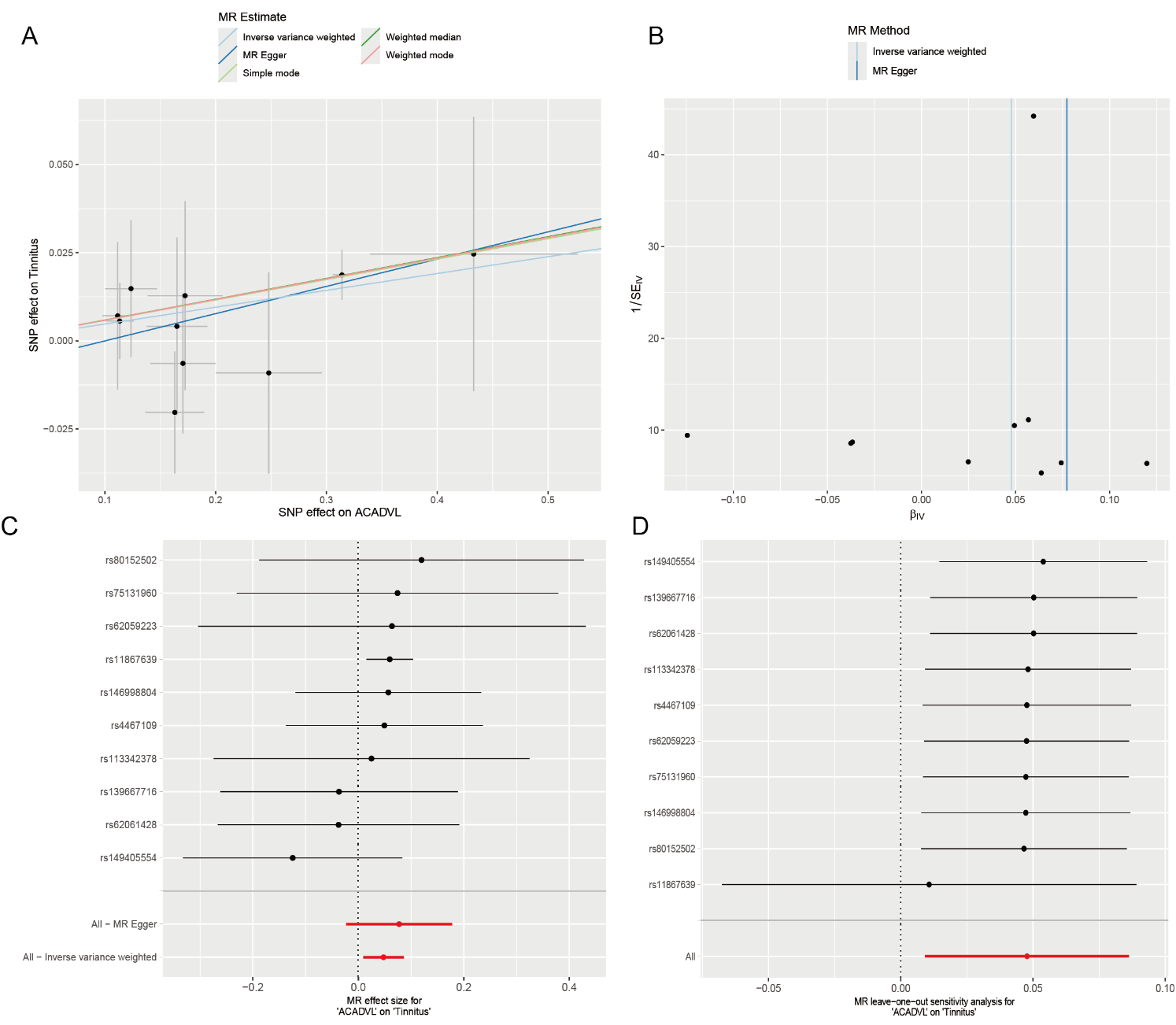


**Supplementary Figure 2**. **Mendelian randomization analysis and sensitivity analysis results of ACADVL eQTL on tinnitus risk.**

**A.** Scatter plot, showing the causal effect of ACADVL eQTLs on tinnitus risk. Each point represents a single SNP. The slope of each regression line corresponds to the MR estimate derived from different methods. The causal estimates were calculated using five Mendelian randomization methods, including inverse variance weighted, MR-Egger regression, weighted median, simple mode, and weighted mode. **B.** Funnel plot assessing potential horizontal pleiotropy and heterogeneity, Each point represents a single SNP. The horizontal axis shows the SNP-specific causal estimate (β_IV), and the vertical axis represents its precision (1/SE_IV). The vertical lines indicate the causal estimates derived from the inverse variance weighted (IVW) method and MR-Egger regression. Symmetry of the funnel plot suggests the absence of substantial horizontal pleiotropy. **C.** Forest plot displaying SNP-specific causal estimates, with the red line indicating the combined overall MR effect. Horizontal lines represent 95% confidence intervals.**D.** Leave-one-out analysis, in which each SNP was sequentially removed to evaluate the stability of the overall causal estimate. Black dots indicate MR estimates after excluding one SNP at a time; horizontal lines represent 95% confidence intervals; the red line indicates the overall MR estimate including all SNPs.


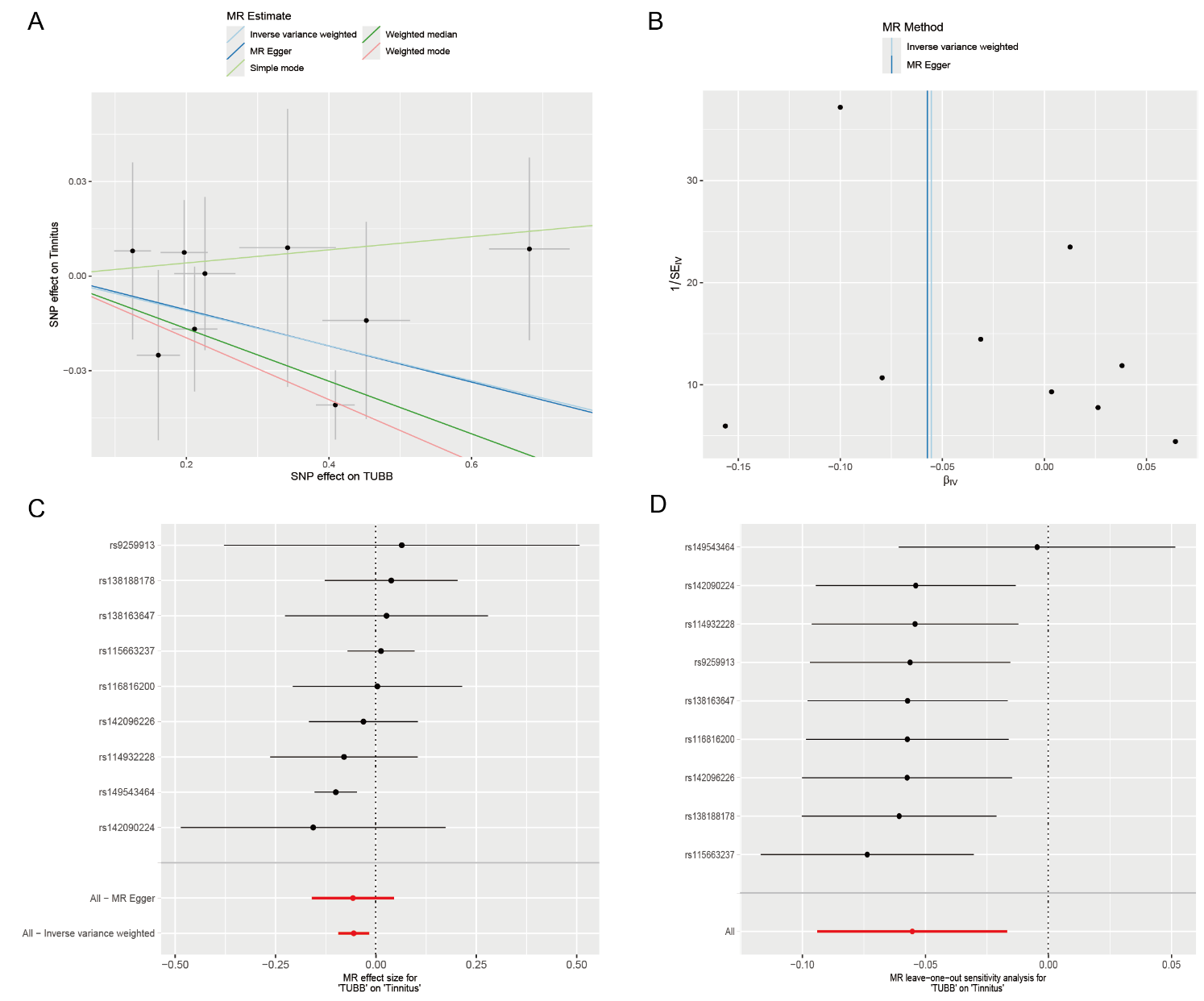


**Supplementary Figure 3**. **Mendelian randomization analysis and sensitivity analysis results of TUBB eQTL on tinnitus risk.**

**A.** Scatter plot, showing the causal effect of TUBB eQTLs on tinnitus risk. Each point represents a single SNP. The slope of each regression line corresponds to the MR estimate derived from different methods. The causal estimates were calculated using five Mendelian randomization methods, including inverse variance weighted, MR-Egger regression, weighted median, simple mode, and weighted mode. **B.** Funnel plot assessing potential horizontal pleiotropy and heterogeneity, Each point represents a single SNP. The horizontal axis shows the SNP-specific causal estimate (β_IV), and the vertical axis represents its precision (1/SE_IV). The vertical lines indicate the causal estimates derived from the inverse variance weighted (IVW) method and MR-Egger regression. Symmetry of the funnel plot suggests the absence of substantial horizontal pleiotropy. **C.** Forest plot displaying SNP-specific causal estimates, with the red line indicating the combined overall MR effect. Horizontal lines represent 95% confidence intervals. **D.** Leave-one-out analysis, in which each SNP was sequentially removed to evaluate the stability of the overall causal estimate. Black dots indicate MR estimates after excluding one SNP at a time; horizontal lines represent 95% confidence intervals; the red line indicates the overall MR estimate including all SNPs.
